# Topoisomerase II minimizes DNA entanglements by proofreading DNA topology after DNA strand passage

**DOI:** 10.1093/nar/gkt1037

**Published:** 2013-10-31

**Authors:** Belén Martínez-García, Xavier Fernández, Ofelia Díaz-Ingelmo, Antonio Rodríguez-Campos, Chaysavanh Manichanh, Joaquim Roca

**Affiliations:** Instituto de Biología Molecular de Barcelona (IBMB), Consejo Superior de Investigaciones Científicas (CSIC), Barcelona 08028, Spain

## Abstract

By transporting one DNA double helix (T-segment) through a double-strand break in another (G-segment), topoisomerase II reduces fractions of DNA catenanes, knots and supercoils to below equilibrium values. How DNA segments are selected to simplify the equilibrium DNA topology is enigmatic, and the biological relevance of this activity is unclear. Here we examined the transit of the T-segment across the three gates of topoisomerase II (entry N-gate, DNA-gate and exit C-gate). Our experimental results uncovered that DNA transport probability is determined not only during the capture of a T-segment at the N-gate. When a captured T-segment has crossed the DNA-gate, it can backtrack to the N-gate instead of exiting by the C-gate. When such backtracking is precluded by locking the N-gate or by removing the C-gate, topoisomerase II no longer simplifies equilibrium DNA topology. Therefore, we conclude that the C-gate enables a post-DNA passage proofreading mechanism, which challenges the release of passed T-segments to either complete or cancel DNA transport. This proofreading activity not only clarifies how type-IIA topoisomerases simplify the equilibrium topology of DNA in free solution, but it may explain also why these enzymes are able to solve the topological constraints of intracellular DNA without randomly entangling adjacent chromosomal regions.

## INTRODUCTION

**Figure 1.**
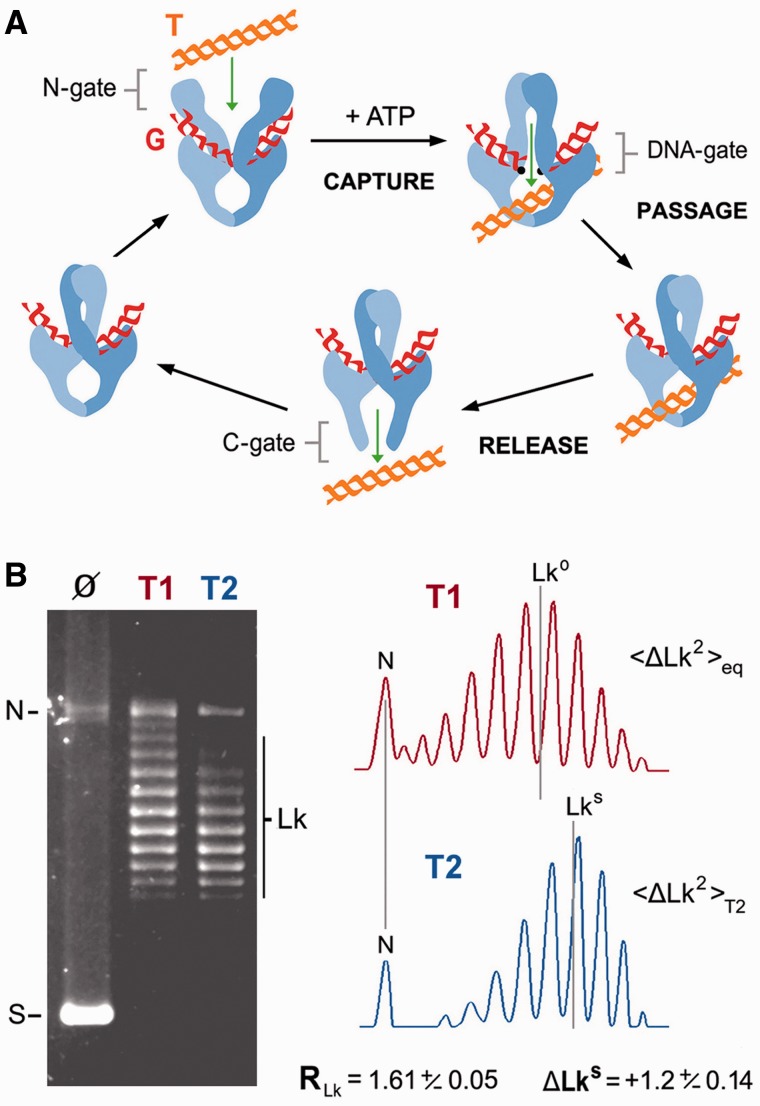
DNA transport mechanism and DNA supercoil simplification activity of topoisomerase II. (**A**) General structure and main steps of the DNA transport mechanism: T-segment capture at the N-gate, T-segment passage across the DNA-gate and T-segment release by the C-gate. CTDs, which are dispensable for DNA transport activity, are not shown. (**B**) Steady-state *Lk* distributions produced by vaccinia virus topoisomerase I (T1) and *S. cerevisiae* topoisomerase II (T2) after relaxation of a negatively supercoiled 7.9-kb plasmid (Ø). The reactions (enzyme/DNA molar ratio of 0.5:1, during 30 min at 37°C), DNA electrophoresis and analysis of *Lk* distributions were done as detailed in the ‘Materials and Methods’ section. The gel position of supercoiled (S), nicked (N) and *Lk* topoisomers (Lk) is indicated. Lane plots compare the variance (R_Lk_ = <Lk^2^>_eq_/<Lk^2^>_T2_) and the central value (Δ*Lk^S^* = *Lk^S^ − Lk^0^*) of both *Lk* distributions. Mean ± SD values of R_Lk_ and Δ*Lk^S^* are from three experiments.

Type-IIA topoisomerases (type-IIA) invert DNA crossovers by transporting one double helix (T-segment) through the transient double-strand break that they produce in another (G-segment) (1). Studies over the past two decades have provided a general picture of type-IIA structure and mechanism (Figure 1A). Type-IIA are homodimers of four functional domains: the ATP-ase domains or N-gate, the DNA cleavage-rejoining core or DNA-gate, the hinge domain or C-gate and the less-conserved C-terminal domains (CTDs) (1,2). To catalyze DNA transport, a G-segment binds first to the cleavage-rejoining core to configure the DNA-gate (3). Binding of ATP causes the ATPase domains to dimerize, and when this closure of the N-gate leads to the capture of a T-segment, a cascade of conformational changes ensues (3). The T-segment is moved toward the DNA-gate, where the G-segment is transiently cleaved by means of transesterification reactions with a pair of symmetrically related tyrosine residues (4). On aperture of the DNA-gate, the passing T-segment reaches the central chamber of the enzyme and it is then released outside the complex by crossing the C-gate (5,6). ATP hydrolysis starts during T-segment transport and concludes to allow N-gate reopening and enzyme turnover (7,8).

Despite using the same general mechanism, distinct type-IIA topoisomerases (bacterial DNA gyrase, bacterial topo IV, eukaryotic topo II) differ in their DNA transport preferences. DNA gyrase selectively introduces (−) supercoils into DNA (9). To do this, the CTDs of gyrase wrap DNA to juxtapose contiguous G- and T-segments and enforce the inversion of (+) supercoil crossings (10). Topo II and topo IV do not have DNA supercoiling activity. They reduce instead the DNA crossovers found in catenaned, knotted and supercoiled DNA molecules (11,12). The CTDs of topo II and topo IV are not required for DNA transport activity but enable different kinetics to unlink right- and left-handed interwinding of DNA duplexes (13,14).

In 1997, Rybenkov *et al.* (15) discovered that topo II and topo IV are able to produce steady-state fractions of catenane, knot and supercoil crossings that are many times lower than the corresponding equilibrium fractions. This directionality of DNA transport has puzzled scientists ever since because it was enigmatic how T-segments could be selected locally to simplify the equilibrium topology of much larger DNA molecules (15,16). Several theories have been proposed. The active sliding model postulated that the topoisomerase forms and actively shortens a DNA loop to corral potential T-segments (15). The kinetic proofreading model proposed that DNA capture and transport requires two successive collisions with potential T-segments. As a result, DNA transport probability has a quadratic relationship with the DNA collision rate (17). The G-segment hairpin model postulated that the topoisomerase produces a sharp bend in the G-segment, which funnels T-segments to be transported preferentially from the inside to the outside of the bend (18). The three-segment interaction model proposed the enzyme reduces the effective DNA contour length because it can interact simultaneously with two potential T-segments before capturing one of them (19). The inter-hooked DNA juxtaposition model postulated that the topoisomerase recognizes the geometry of interhooked DNA crossovers as potential G- and T-segments (20). Thus far, experimental results have discarded the sliding mechanism (16) and ruled out the kinetic proofreading (21). Only the hairpin model is backed by the experimental evidence of a strong bend induced by type-IIA topoisomerases in the G-segment (18,22). However, this bend is not sufficiently sharp to explain the degree of DNA topology simplification experimentally observed (18,23). Recent studies have also shown that bending angles produced by different type-IIA do not correlate with the simplification efficiency of the corresponding enzymes (24,25). Thus, the mechanism by which type-IIA simplifies equilibrium DNA topology remains controversial and its biological relevance poorly understood.

Here we examined the transit of the T-segment across the three gates of yeast topoisomerase II during the simplification of equilibrium fractions of DNA supercoils. Our results uncovered that simplification of equilibrium DNA topology occurs because, after T-segment capture and passage, the topoisomerase challenges the release of the T-segment with the C-gate, and that this constriction permits either the completion or backtracking of DNA transport. This post-DNA passage proofreading activity not only clarifies how type-IIA simplifies equilibrium DNA topology, but also suggests a crucial biological role by preventing the random entangling of intracellular DNA.

## MATERIALS AND METHODS

### DNA and topoisomerases

Plasmids pBR322 (4.3 kb) and YCp50 (7.9 kb) were purified by density gradient centrifugation in cesium chloride following standard procedures. Topoisomerase I of vaccinia virus (T1) was produced in *Escherichia coli* and purified as previously described (26). A CTD-less topoisomerase II of *Saccharomyces cerevisiae* (T2) was produced from pGAL1Top2(1196)-HMK-His (27) in the protease-deficient yeast strain BCY123-*Δtop1*. This strain was constructed by disrupting the *TOP1* gene in BCY123, as previously described (28). T2 was purified following the procedures previously described for the full-length yeast topo II (4). T2 was stored at a concentration of 2 mg/ml at −80°C, and working stocks at 100 ng/ml were kept at −20°C in 50 mM Tris-HCI (pH 8), 1 mM ethylenediaminetetraacetic acid (EDTA), 500 mM KCI, 7 mM 2-mercaptoethanol, 100 µg/ml bovine serum albumin (BSA) and 50% (v/v) glycerol. The T2 derivative, in which the C-gate can be reversibly locked by a pair of engineered disulfide bonds, was obtained by mutating two amino residues, N1043C and K1127C, as previously described (6). This enzyme was produced and kept under the same conditions as T2, but omitting sulfhydryl reagents in all buffers.

### DNA relaxation and analysis of *Lk* distributions

DNA plasmids (0.2 pmol) were incubated with T1(catalytic excess) or T2 (at the specified molar ratio) in a 50-μL volume of 50 mM Tris–HCl (pH 8), 1 mM EDTA, 150 mM KCl, 8 mM MgCl_2_, 7 mM 2-mercaptoethanol and 100 µg/ml BSA. T2 reactions were initiated by the addition of ATP (1 mM) when indicated. Temperature and incubation times are specified in each experiment. Reactions were stopped with the addition of 20 mM EDTA, 0.5% (w/v) sodium dodecyl sulphate (SDS) and 100 μg/ml proteinase K, and incubated for 15 min at 50°C. Reaction samples were loaded onto 0.8% (w/v) agarose gels. DNA electrophoresis was carried out at 1.6 V/cm for 18 h in TBE buffer (50 mM Tris-borate, 1 mM EDTA) containing 0.2 μg/ml chloroquine. In these conditions, the distributions of *Lk* topoisomers of relaxed DNA circles adopt positive writhe and migrate faster than nicked circles. Gels were stained with ethidium bromide, destained in water and photographed over an ultraviolet light source with a Kodak GL1500 camera. Plots of *Lk* distributions and quantification of *Lk* topoisomers were done using Kodak Molecular Imaging Software v4.5 and Image 1.34 s. The topoisomer variance of *Lk* distributions <Δ*Lk*^2^> was calculated as Σ[*pi*(Δ*Lki*)^2^]/Σ*pi*, where *pi* is the amount of each topoisomer *i* in the distribution and Δ*Lki* is the linking number for topoisomer *i* relative to that of a reference topoisomer near the center of the distribution.

### T-segment capture in single *Lk* topoisomers

Individual *Lk* topoisomers of pBR322 (4.3 kb) were purified from unstained agarose gel slices. Approximately 50 fmol of DNA topoisomer and 25 fmol of T2 were mixed at 25°C in a 25 -μL volume containing 50 mM Tris–HCl (pH 8), 1 mM EDTA, 150 mM KCl, 8 mM MgCl_2_, 7 mM 2-mercaptoethanol and 100 mg/ml BSA. AMPPNP was added to 2 mM to close the N-gate and form high salt-resistant T2/DNA complexes (3). After 5 min of incubation, 1 volume of 2 M NaCl was added, and the mixture was passed through a GF/C (Whatman) fiberglass filter as described in (29). Free DNA molecules were recovered from the filtrate, and T2-bound DNA circles were eluted from the filter with 1% SDS. Both fractions were analyzed by electrophoresis (2 V/cm for 14 h) in 1% agarose in TBE buffer (50 mM Tris-borate, 1 mM EDTA) containing 0.2 μg/ml chloroquine. DNA populations were quantified by phosphor-imaging analysis of the gel-blot probed with ^32^P-labeled DNA obtained by random priming.

### T-segment backtracking in single *Lk* topoisomers

Approximately 50 fmol of gel-purified *Lk_i_* topoisomer and 25 fmol of the T2 derivative, in which the C-gate was locked by a pair of disulfide bonds, were mixed at 15°C in a 40 -μL volume of 50 mM Tris–HCl (pH 8), 1 mM EDTA, 25 mM KCl, 8 mM MgCl_2_ and 100 mg/ml BSA. The mixture was supplemented either with ATP (1 mM) or AMPPNP (2 mM) and incubated for 5 min. Half of the mixture (20 µL) was then shifted to 40°C. After 5 min of incubation, both reactions were quenched with 1 volume of 50 mM EDTA, 1% (w/v) SDS. DNA products were analyzed by electrophoresis in 1% agarose gel in TBE buffer (50 mM Tris-borate, 1 mM EDTA) containing 0.2 μg/ml chloroquine. DNA populations were quantified by phosphor-imaging analysis of the gel-blot probed with ^32^P-labeled DNA obtained by random priming.

### Construction of T2Δ83

Plasmid pGAL1Top2(1196)-HMK-His was used as polymerase chain reaction template to generate an 83-amino acid deletion between Leu1039 and Trp1122 of the *TOP2* gene. Primer Δ83-Fwd, 5′ *GCGGGT*TGGTCATTGACCAAGGAAAG 3′, was complement to the 3367–3386 *TOP2* gene fragment and had a 5′-six nt tail (italicized) coding for Ala-Gly. Primer Δ83-Rev, 5′ *GGCCGC*TAACTCCTTTTCAATAATCA 3′, was reverse complement to the 3098–3117 *TOP2* gene fragment and included a 5′-six nt tail (italicized) coding for Ala-Ala. Both primers were extended in a thermal cycler by *Pfu* DNA polymerase. The reaction products were digested with *Dpn*I endonuclease to degrade the initial template, and the amplified DNA was transformed into *E. coli* DH5α electrocompetent cells. Transformants were screened for those containing the religated DNA plasmid that substituted the 83 amino acids by the spacer sequence Ala-Ala-Ala-Gly, which gave rise to unique *Not*I and *Sac*II restriction sites. The modification was confirmed by DNA sequencing. The new plasmid, pGALT2Δ83HMK-His, was introduced in the BCY123-*Δtop1* yeast strain to overexpress and purify T2Δ83, following the procedure described in (4).

## RESULTS

### Simplified distributions of DNA supercoils produced by topoisomerase II

Figure 1B compares the distributions of DNA linking number topoisomers (*Lk*) obtained after incubating a supercoiled DNA plasmid with topoisomerase I of vaccinia virus (T1) and topoisomerase II of *S. cerevisiae* (T2). As expected, T2 generated an *Lk* distribution, the variance <Δ*Lk^2^*> _T2_ of which was smaller than that of the thermal equilibrium *Lk* distribution <Δ*Lk^2^*>_eq_ produced by T1 in the same reaction conditions. The parameter R_Lk_, defined as <Δ*Lk^2^*>_eq_/<Δ*Lk^2^*> _T2_, was ∼1.6. Because the center of the non-equilibrium *Lk* distribution generated by T2 (*Lk^S^*) did not always coincide with the equilibrium center (*Lk^0^*) produced by T1, *Lk^S^–Lk^0^* was defined as Δ*Lk^S^.* This simplification activity of T2 is efficient and robust. With T2/DNA molar ratios of 1:1, a negatively supercoiled 7.9-kb plasmid was relaxed and its thermal *Lk* distribution narrowed (R_Lk_∼1.6) in ∼1 min (Supplementary Figure S1A). Similar R_Lk_ values were achieved in a broad range of salt concentrations, from 10 to 250 mM KCl (Supplementary Figure S1B), and reaction temperatures, from 10 to 45°C (Supplementary Figure S1C). However, the symmetry of the narrowing process (Δ*Lk^S^*) highly depends on the reaction temperature*.* At 25°C, Δ*Lk^S^∼0*; at higher temperature, Δ*Lk^S^>0*; and, at lower temperature, Δ*Lk^S^<0*. Thus, thermal changes do not deviate *Lk^S^* as much as they deviate *Lk^0^* (Supplementary Figure S1C).

### Supercoil simplification is not determined by T-segment capture

**Figure 2.**
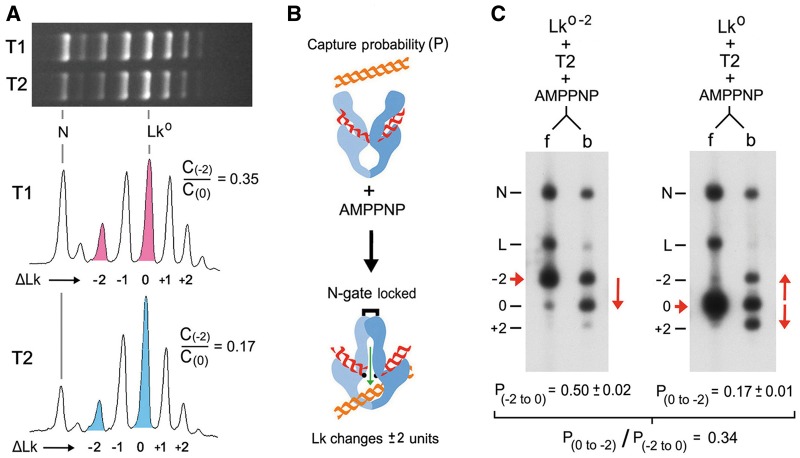
Interconversion of *Lk* topoisomers and their T-segment capture probability. (**A**) *Lk* distributions produced by T1 and T2 on a 4.3-kb plasmid at 25°C. Lane plots compare the concentration (C) of topoisomers *Lk^0^^−^^2^* to *Lk^0^* in the equilibrium distribution generated by T1 (0.35) and in the non-equilibrium distribution produced by T2 (0.17). (**B**) A T2 enzyme bound to a G-segment will capture and pass a T-segment with some probability after the irreversible closure of the N-gate with AMPPNP. Thus, in a covalently closed DNA circle, DNA capture probability (P) is the fraction of G-segment-bound DNA molecules that change *Lk* (2 U) on addition of AMPPNP. (**C**) Topoisomers *Lk^0^^−^^2^* and *Lk^0^* were purified and each was incubated at 25°C with T2 (enzyme/DNA molar ratio of 0.5:1). AMPPNP was added to irreversibly close the N-gate and thus allow only one DNA passage event per enzyme. Reaction mixtures were filtered through glass fibers to separate free DNA (f) from DNA circles bound to T2 (b). See ‘Materials and Methods’ section for details. The gel-blots show the DNA populations in f and b. The position of *Lk^0^^−^^2^, Lk^0^* and *Lk^0+2^* is indicated (−2, 0, +2). Nicked circles (N) and linear molecules (L) were generated during manipulation. The probability of capturing a T-segment to convert *Lk^0–2^* into *Lk^0^* [P_(−2 to 0)_] was calculated as *Lk^0^* divided by the sum of circles bound to T2 (*Lk^0−2^+Lk^0^*). Likewise, the probability of capturing a T-segment to convert *Lk^0^* into *Lk^0−2^* [P_(0 to −2)_] was calculated as *Lk^0−2^* divided by the sum of circles bound to T2 (*Lk^0^ + Lk^0−2^ + Lk^0+2^*). Mean ± SD values are from two experiments.

Figure 2A shows the *Lk* distributions produced by T1 and T2 on a 4.3-kb plasmid at 25°C. Because type-IIA topoisomerases change *Lk* in steps of two, the *Lk* distribution generated by T2 is in fact composed of two independent *Lk* distributions, one of odd values and one of even values. Therefore, topoisomers *Lk^0^* and *Lk^0^^−^^2^* have steady-state concentrations (*C_0_* and *C_-2_*) related by the equation *C_−__2_*/*C_0_ = k_(0,__−_* *_2)_/k_(__−__2_, _0)_*, where *k_(0, __−__2)_* is the rate constant for conversion of *Lk^0^* molecules into *Lk^0^^−^^2^*, and *k_(__−__2_, _0)_* is the reverse rate constant (23,30). As expected, T2 activity produced a *C_−__2_*/*C_0_* ratio (0.17) much lower than that generated by T1 (0.35). To reduce *C_−__2_*/*C_0_*, T2 must decrease *k_(0, __−__2)_/k _(__−__2_, _0)_*. In a covalently closed DNA circle, any DNA transport event done by T2 results in a change in *Lk*. Therefore, rate constants *k_(0, __−__2)_* and *k_(__−__2_, _0)_* may relate directly to the corresponding probability (*P*) of capturing and passing a T-segment across the G-segment. It would then follow that *C_−__2_*/*C_0_* = *P_(0, __−__2)_*/*P_(__−__2_, _0)_*. Later, we present experimental evidence that disproves this equality.

Biochemical studies have shown that a T-segment cannot be accommodated between the closed N-gate and the DNA-gate of T2 (31). This restraint is consistent with the swapped configuration of the closed N-gate (32), which enforces unidirectional passage of the T-segment across the DNA-gate following the capture step. Consequently, *P_(0, __−__2)_* and *P_(__−__2_, _0)_* can be calculated by examining the fractions of *Lk^0^* and *Lk^0^^−^^2^* bound to T2 that interconvert after the irreversible closure of the N-gate with the non-hydrolysable ATP analog AMPPNP (Figure 2B) (33). Thus, we purified the *Lk^0^* and *Lk^0^^−^^2^* topoisomers and conducted these one-step reactions in the same conditions used to calculate *C_0_* and *C_−__2_*. We found that the probability of capturing a T-segment to convert *Lk^0^* into *Lk^0^^−^^2^* was 0.17, whereas the probability to convert *Lk^0^^−^^2^* into *Lk^0^* was 0.50 (Figure 2C). These values predicted a *P_(0, __−__2)_/P_(__−__2_, _0)_* ratio of 0.34. This value was similar to the *C_−__2_*/*C_0_* ratio at thermal equilibrium (0.35) and thus differed from the steady-state *C_−__2_*/*C_0_* ratio generated by T2 (0.17). Therefore, the mechanism that narrows the equilibrium distributions of DNA supercoils does not rely on T-segment capture probability.

### Passed T-segment can backtrack across the DNA-gate and N-gate

Once the captured T-segment has crossed the DNA-gate, it can be accommodated in the central cavity of the topoisomerase (6,31). To complete DNA transport, the passed T-segment has to be expelled by the C-gate. Otherwise, if the N-gate reopens or loosens its swapped configuration, the T-segment could still backtrack across the DNA-gate and produce no net DNA transport. This uncoupling between T-segment capture and transport has been observed in DNA gyrase when the supercoiling density of DNA reaches a threshold (10). Later, we present experimental evidence that such backtracking can also occur in T2.

**Figure 3.**
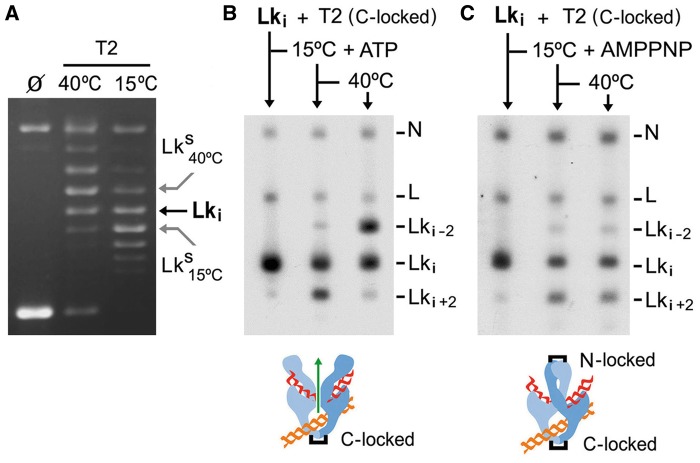
Backtracking of the T-segment across the DNA-gate and N-gate. (**A**) The gel compares *Lk* distributions of a 4.3-kb plasmid generated by T2 at 15 and 40°C. The position of the *Lk_i_* topoisomer is indicated. Note that *Lk_i_*<*Lk^S^* at 15°C and *Lk_i_*>*Lk^S^* at 40°C. (**B**) The gel-blot shows the purified *Lk_i_* topoisomer and its reaction with T2, which had the C-gate locked (enzyme/DNA molar ratio of 0.5:1). The mixture was first incubated at 15°C during 5 min in the presence of ATP and sampled. The mixture was then shifted to 40°C for 5 more minutes and sampled. See ‘Materials and Methods’ section for details. (**C**) Experiment done as in (B), but in the absence of ATP and with AMPPNP added at 15°C. In this way, both the C-gate and N-gate were locked after T-segment passage. The position of topoisomers *Lk_i-2_*, *Lk_i_* and *Lk_i+2_* is shown. Nicked circles (N) and linear molecules (L) were generated during manipulation.

Previous studies have shown that when the C-gate of T2 is locked by means of engineered disulfide bonds, each enzyme bound to supercoiled DNA can change *Lk* only by 2 U because any T-segment passed ends up entrapped inside the topoisomerase (6,31). In those experiments, DNA supercoiling energy favored unidirectional movement of the T-segment, and backtracking was not observed. Here, we conducted a similar experiment but with relaxed DNA. To test backtracking, we purified a precise topoisomer (*Lk_i_*) within the simplified *Lk* distribution produced by T2. *Lk_i_* was chosen because *Lk_i_*<*Lk^S^* at 15°C and *Lk_i_*>*Lk^S^* at 40°C (Figure 3A). In this way, we could invert the preferential directionality of a T-segment across the DNA-gate by changing the reaction temperature from 15 to 40°C. Accordingly, we first incubated *Lk_i_* with T2 (with the C-gate locked and in presence of ATP) at 15°C. As expected, a fraction of *Lk_i_* was converted into *Lk_i+2_*. Next, we raised the temperature to 40°C. As a result, the *Lk_i+2_* fraction nearly disappeared and a fraction of *Lk_i__−__2_* molecules developed (Figure 3B). The disappearance of the *Lk_i+2_* revealed the backtracking of T-segments entrapped at 15°C. Likewise, the appearance of *Lk_i__−__2_* indicated that such T-segments had escaped by the N-gate. Only by this way could new T-segments have been captured and passed at 40°C to produce the population of *Lk_i__−__2_*. We corroborated that backtracking requires the opening of the N-gate by doing an analogous experiment, in which we added AMPPNP instead of ATP. In this case, *Lk_i_* were also converted into *Lk_i+2_* at 15°C. However, because AMPPNP did not allow the N-gate to reopen, no more *Lk* changes occurred when the temperature was raised to 40°C (Figure 3C).

### Simplified *Lk* distributions widen when T-segment backtracking is precluded

**Figure 4.**
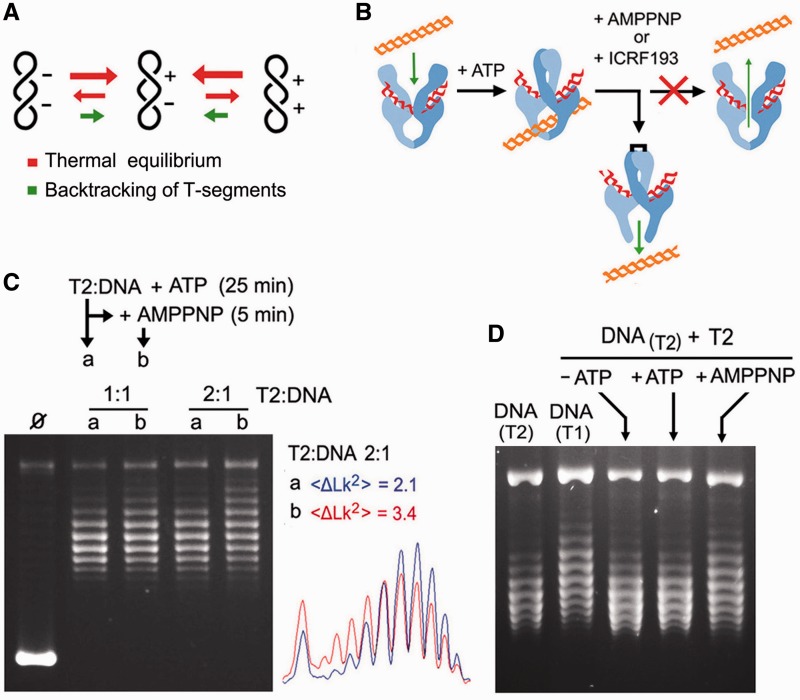
Simplified *Lk* distributions widen when T-segment backtracking is precluded. (**A**) We hypothesize that the narrowing of *Lk* distributions occurs because T-segments that deviate *Lk* from equilibrium are those more prone to backtrack. (**B**) Compounds that block the reopening of the N-gate (AMPPNP or ICRF-193) impede DNA backtracking, and therefore they should interfere with the simplification activity of T2 during steady state. (**C**) A 7.9-kb supercoiled DNA plasmid was incubated with T2 (T2/DNA molar ratios of 1:1 and 2:1) in the presence of ATP (0.1 mM) at 30°C. After 25 min, half of each reaction was sampled (lane a), and AMPPNP (2 mM) was added to the other half for 5 more minutes (lane b). Gel plots compare the variance of *Lk* distributions (<Δ*Lk^2^*>) before and after the addition of AMPPNP (T2/DNA molar ratio of 2:1). (**D**) Lanes DNA_(T1)_ and DNA_(T2)_ show the *Lk* distributions of a 7.9-kb plasmid produced by T1 and T2, respectively. The DNA_(T2)_ sample was purified and incubated again with T2 (T2/DNA molar ratio of 2:1) during 5 min in the absence of ATP, in the presence of ATP (1 mM) and in the presence of AMPPNP (2 mM). Reaction settings, gel electrophoresis and analyses of *Lk* populations were as described in the ‘Materials and Methods’ section.

The above results indicated that backtracking of passed T-segments is possible when DNA topology is near equilibrium. Accordingly, we envisaged that T2 narrows equilibrium *Lk* distributions because passed T-segments driving *Lk* away from equilibrium are more prone to backtrack than those driving *Lk* toward it (Figure 4A). If this hypothesis is correct, interfering with the reopening of the N-gate while T2 is narrowing an *Lk* distribution, it should broaden the *Lk* distribution because none of the T-segments being passed at that time would be able to backtrack (Figure 4B). Thus, we relaxed a supercoiled plasmid with T2 in the presence of ATP. Once the reaction had reached the steady state, we blocked the reopening of the N-gate by adding an excess of AMPPNP over the initial ATP concentration. As seen in Figure 4C, the simplified *Lk* distribution generated by T2 readily increased its variance on the addition of AMPPNP. This broadening cannot be attributed to contaminating topo I because T2 was purified from Δ*top1* cells, and no trace of DNA relaxation activity was observed in the absence of ATP. Likewise, this broadening was unlikely to be produced by the structure of T2/AMPPNP complexes, as previous studies indicated that they do not significantly alter the topology of the interacting DNA (19). Yet, because each T2/AMPPNP complex precluded at most one backtracking event, this effect had to be stoichiometric and, effectively, the widening increased with the molar ratio of T2 to DNA. Note also that the widening was not symmetric. This shift occurred because *Lk^S^>Lk^0^* at 30°C (Supplementary Figure S1C). Accordingly, most DNA passage events displaced the non-equilibrium distribution (centered in *Lk^S^*) toward the equilibrium distribution (centered in *Lk^0^*). Remarkably, an identical result was obtained by blocking the reopening of the N-gate in a different way. Instead of adding AMPPNP to compete with ATP, we added the N-gate inhibitor ICRF193 (34,35) (Supplementary Figure S2).

To further verify that supercoil simplification depends on the backtracking of passed T-segments that drive *Lk* away from equilibrium, we conducted a similar experiment but using as initial DNA substrate a purified *Lk* distribution previously narrowed by T2. As seen in Figure 4D, the simplified *Lk* distribution was not altered after T2 incubation, either in the absence or presence of ATP. However, it clearly broadened when incubation with T2 was followed by the addition of AMPPNP. As expected, without possible backtracking of passed T-segments, most DNA passage events tended to convert the non-equilibrium *Lk* distribution into the thermal equilibrium distribution produced by T1.

### C-gate integrity is required to simplify equilibrium *Lk* distributions

**Figure 5.**
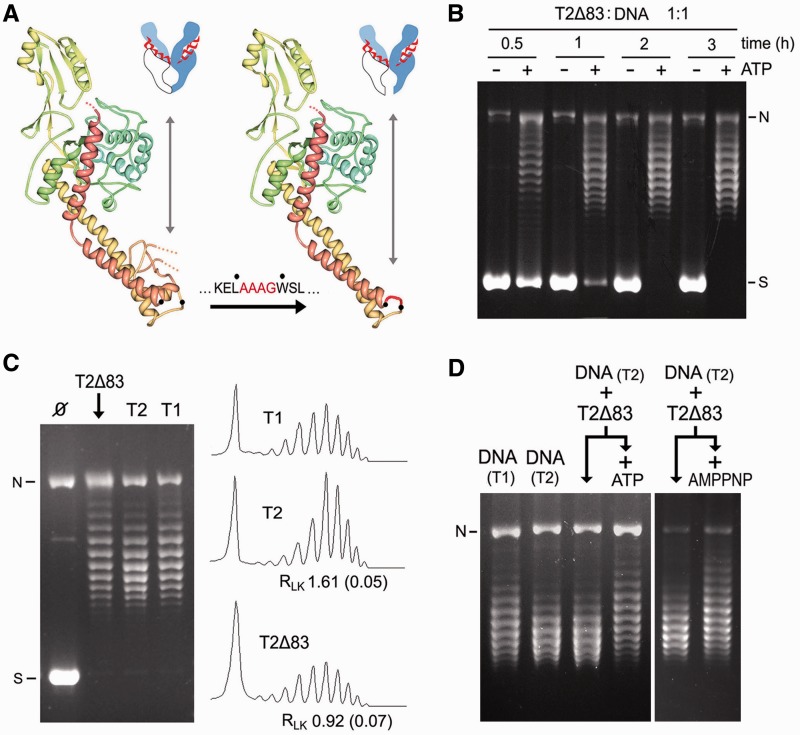
Disruption of the C-gate precludes the simplification of equilibrium DNA topology. (**A**) Structure of the promoter region that configures the central chamber of T2 and the replacement of 83 amino residues of the C-gate domain, between L1039 and W1122, by an AAAG linker (T2Δ83). (**B**) Time course relaxation of a negatively supercoiled DNA plasmid (7.9 kb) with T2Δ83 (E/DNA molar ratio of 1, 37°C) in the absence and presence of ATP (1 mM). (**C**) Comparison of the steady-state *Lk* distributions produced by T2Δ83, T2 and T1 (E/DNA molar ratio of 1:1, 37°C, 6 h). Plots of the *Lk* distributions and mean R_Lk_ (SD) values of from three experiments are shown. (**D**) Lanes DNA_(T1)_ and DNA_(T2)_ show *Lk* distributions produced by T1 and T2. The DNA_(T2)_ sample was purified and incubated with T2Δ83 (E/DNA molar ratio of 1:1, 37°C, 30 min) in the absence and presence of ATP (1 mM). The DNA_(T2)_ sample was also incubated with T2Δ83 (E/DNA molar ratio of 5:1, 37°C, 30 min) alone or following the addition of AMPPNP (2 mM). Reaction settings, gel electrophoresis and analyses of *Lk* populations were as described in the ‘Materials and Methods’ section. N, nicked circles. S, supercoiled DNA.

The mechanism for topology simplification inferred from the above results predicted another experimental outcome: if the C-gate were deleted to leave the central chamber of T2 open, all passed T-segments would readily exit the enzyme and produce net transport. Consequently, topology simplification that relies on DNA backtracking would not occur. To test this hypothesis, we engineered a T2 polypeptide chain (T2Δ83), in which 83 amino residues of the C-gate of T2 (36), between L1039 and W1122, were substituted by a short residue linker (AAAG) (Figure 5A). T2Δ83 was produced in the yeast strain BCY123-*Δtop1* and purified to test its activity (Supplementary Figure S3). Filter-binding assays (29) revealed that, although T2Δ83 binds DNA, it does not form high salt-stable complexes with circular DNA following AMPPNP addition (Supplementary Figure S4). This observation corroborated that the disruption of the C-gate lets the central chamber of the enzyme permanently open and, hence, it cannot produce a toroid around DNA on closure of the N-gate.

T2Δ83 relaxed supercoiled DNA in an ATP-dependent manner (Figure 5B). However, the specific activity of T2Δ83 was near two orders of magnitude below that of T2 because it required a 2-h incubation at an enzyme/DNA molar ratio of 1:1 to produce steady-state *Lk* distributions (Figure 5B). This reduced activity was expected, as the global stability of the enzyme could be altered and its interdomain couplings could be less efficient in the absence of the C-gate interface. When we compared the steady-state *Lk* distributions produced by T2Δ83, T2 and T1 in the same reaction conditions and longer incubation times (6 h), we found that T2Δ83 was not able to simplify DNA topology to below equilibrium (Figure 5C). This outcome could not be attributed to contaminating topo I because T2Δ83 was purified from Δ*top1* cells. To further corroborate that simplified *Lk* distributions were not the final product of T2Δ83 activity, we use as initial DNA substrate of the relaxation reaction an *Lk* distribution previously narrowed by T2. As shown in Figure 5D, incubation of the simplified *Lk* distribution with T2Δ83 in the absence of ATP produced no changes. However, in the presence of ATP, T2Δ83 broadened and shifted the *Lk* distribution into a shape similar to that produced by T1. Therefore, the C-gate integrity was not essential for relaxing DNA supercoils but it was required for narrowing *Lk* distributions to below the equilibrium values.

Given that T2Δ83 has the C-gate permanently open, it is conceivable that this enzyme may be able to conduct DNA transport in reverse. However, when a simplified *Lk* distribution was incubated with T2Δ83 and AMPPNP was added (instead of ATP), a broadening effect was also observed (Figure 5D, right). Because a T-segment cannot be held between the DNA-gate and the closed N-gate, this observation argued against DNA passage in reverse. Yet, it cannot be fully discredited that a T-segment enters by the open C-gate, crosses the DNA-gate and then exits by the N-gate before it closes on nucleotide binding.

## DISCUSSION

The experiments reported here uncover several new traits of the DNA transport mechanism of topoisomerase II. First, T-segment capture probability is determined mostly by DNA thermodynamics. Therefore, the mechanism that simplifies equilibrium DNA topology must operate after the capture of the T-segment. Second, once a passed T-segment has reached the central chamber of the topoisomerase, it is able to backtrack across the DNA-gate and N-gate, thus reverting DNA transport. This possibility elucidates why T-segment capture is not the sole determinant of DNA transport probability. This notion of T-segment backtracking is not entirely new, as it has been also observed in DNA gyrase (10). Third, simplification of equilibrium DNA topology does not occur when the backtracking of T-segments is prevented. We supported this conclusion in two ways: by blocking the reopening of the N-gate and by removing the C-gate. All these findings strongly suggest that the C-gate of topoisomerase II challenges the release of passed T-segments, and that this restraint allows proofreading DNA topology after T-segment passage, either to complete or to cancel DNA transport.

The factors that control the C-gate of type-IIA are unknown. The C-gate status could depend on the DNA-gate, such that the closure of either gate permits the aperture of the other (37,38). However, crystal images of type-IIA with both gates closed indicate that DNA-gate closure does not strictly enforce C-gate opening (39,40). Thus, C-gate aperture could be triggered by the steric hindrance of the passed T-segment after the DNA-gate closes (31,41). In any case, the exit of the T-segment across the C-gate is a dissociation process, the rate of which must be affected by the molecular environment (i.e. friction and electrostatic protein–DNA interactions) and the global DNA topology (i.e. thermodynamic energy of the crossover of the G-segment with the passed T-segment). Consequently, T-segment dissociation is likely to be fast when DNA transport is energetically favorable, but slow when DNA transport reaches or eventually departs from topology equilibrium. In this last case, if the N-gate reopens before the T-segment has escaped by the C-gate, our results demonstrate that backtracking of the T-segment can occur. We envisaged then that equilibrium DNA distributions are narrowed because passed T-segments that deviate DNA topology from the equilibrium center are those more likely to backtrack. Accordingly, removing the C-gate should increase the dissociation rate of such T-segments and preclude the simplification activity, as demonstrated by our results.

**Figure 6.**
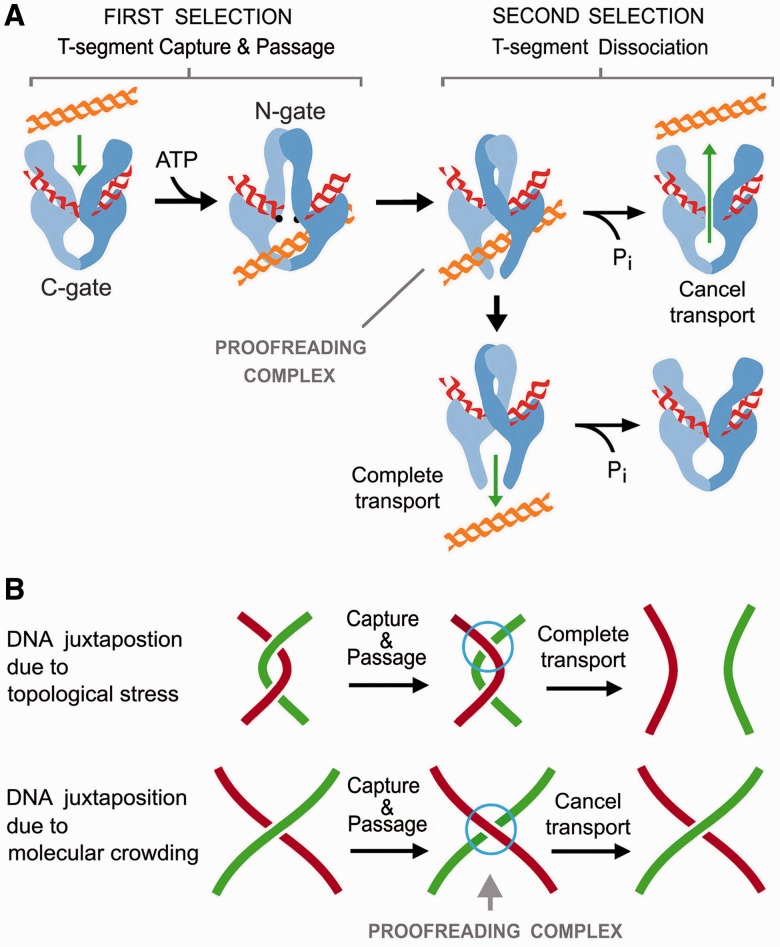
Post-DNA passage proofreading mechanism of topoisomerase II. (**A**) Under PPR, DNA transport probability results from two levels of selection. First, DNA juxtaposition probability determines T-segment capture by the closure of the N-gate on ATP binding. Second, after T-segment capture and passage, the C-gate challenges the T-segment to dissociate from the ‘proofreading complex’. When DNA topology favors quick dissociation, the T-segment crosses the C-gate and completes DNA transport. However, when dissociation is not favored, ATP hydrolysis and reopening of the N-gate occurs before the T-segment has escaped by the C-gate. In this case, the T-segment backtracks and produces no net DNA transport. (**B**) PPR permits the removal of topological constraints generated during DNA transcription and replication (quick dissociation of passed T-segments). PPR prevents the entanglement of independent chromosome domains when their DNA strands come in proximity due to molecular crowding (slow dissociation of passed T-segments).

The simplification of DNA topology to below equilibrium values is an uphill reaction that, like DNA supercoiling by gyrase, could not occur without consuming ATP. However, in contrast to DNA supercoiling by gyrase, the amount of free energy required here is insignificant (<1%) compared with the total free energy available from ATP hydrolysis (16). Actually, all type-IIA consume ATP, regardless of the energetics of the topology interconversions (42,43). These premises have led to the proposal that the main and ancestral role of ATP is the coordination of the enzyme gates to prevent DNA double-strand breaks (41,44). In this regard, our present findings provide additional insight on the role of ATP. Enzymatic proofreading requires some irreversible steps to improve the selectivity of a reaction. The capture of a T-segment by the N-gate and its subsequent release, by either the C-gate or N-gate, are separated by the irreversible use of ATP. Therefore, the ATP cycle of type-IIA enables a proofreading scheme, in which the first step of selection (capture) is either enhanced or neutralized by the second (release). While T-segment capture probability relies mainly on DNA topology, the dissociation rate of the T-segment depends again on DNA topology and the constraints of the C-gate. Therefore, combination of both selection levels can produce an acute non-linear relationship between DNA capture probability and effective DNA transport (Figure 6A).

A striking observation in our study is the effect of temperature on Δ*Lk^S^* (Supplementary Figure S1C). Previous studies postulated that Δ*Lk^S^* could result from three-segment interaction geometry (19). This hypothesis is no longer valid because it fails to explain why *Lk^S^* does not parallel the thermal changes of *Lk^0^*. The reduced thermal deviations of *Lk^S^* observed here suggest that DNA behaves as if its free length were shorter. This shortening effect may reflect the formation of the proofreading complex, which entraps a crossover of the G-segment and the passed T-segment. Thus, the DNA is transiently divided into two smaller thermodynamic domains during the proofreading step.

The biological relevance of the type-IIA capacity to simplify the equilibrium topology of DNA in free solution has been enigmatic because this activity cannot be extrapolated to *in vivo* systems (16). We believe that the post-DNA passage proofreading mechanism (PPR) disclosed here elucidates this matter. Unlike other models of topology simplification (15,17–20), in which DNA transport probability is determined just by T-segment capture, the PPR mechanism weighs the dissociation rate of passed T-segments. Dissociation across the C-gate is likely to be favored when the topoisomerase inverts DNA crossovers generated by topological stress (i.e. catenanes between newly replicated DNA duplexes and supercoils arising during DNA replication and transcription). However, dissociation may be less favored when the topoisomerase inverts transient juxtapositions of intracellular DNA that are merely due to molecular crowding. In this case, PPR can cancel DNA transport and prevent type-IIA topoisomerases from randomly entangling the vast concentration of intracellular DNA (Figure 6B). Thus, PPR may play a fundamental role in preserving the high compartmentalization of chromosomal territories during interphase, and in keeping sister chromatids topologically unlinked even though they are in close contact until anaphase. This crucial discrimination of intracellular DNA juxtapositions could not be possible if DNA transport selectivity of type-IIA were determined solely by T-segment capture probability.

Because all type-IIA topoisomerases that simplify equilibrium DNA topology present high structural conservation of the N-gate, DNA-gate and C-gate (1,2), the PPR mechanism may be a common trait of these enzymes. In this regard, the more distant related family of type-IIB topoisomerases (i.e. archea topo VI) does not have a C-gate to challenge the release of passed T-segments and, remarkably, these enzymes do not simplify DNA topology to below equilibrium values (45).

PPR complements the other mechanisms that control the DNA transport selectivity of type-IIA enzymes *in vivo*. While the geometry of the interacting DNA segments optimizes the removal of harmful knots and catenanes, the PPR mechanism minimizes the random formation of such entanglements in the first place. Uncovering the DNA damage and chromosomal aberrations that result from interfering with the PPR mechanism opens up an interesting area for future research.

## SUPPLEMENTARY DATA

Supplementary Data are available at NAR Online.

## FUNDING

The Plan Nacional de I+D+I of Spain [BFU2008-00366 and BFU2011-23851 to J.R.]; Pla de Recerca de Catalunya [2009SGR01222 to J.R.]; Xarxa de Referencia en Biotecnología de la Generalitat de Catalunya. Funding for open access charge: Plan Nacional de I+D+I of Spain [BFU2011-23851 to J.R.].

*Conflict of interest statement*. None declared.

## Supplementary Material

Supplementary Data
