# Supplementary material for: Topoisomerase II minimizes DNA entanglements by proofreading DNA topology after DNA strand passage
Source: Nucleic Acids Res. 2013 Oct 31;42(3):1821–30. doi: 10.1093/nar/gkt1037 (PMC3919613; doi:10.1093/nar/gkt1037)
Supplement: Supplementary Data [file supp_42_3_1821__index.html]

Topoisomerase II minimizes DNA entanglements by proofreading DNA topology after DNA strand passage — Topoisomerase II minimizes DNA entanglements by proofreading DNA topology after DNA strand passage — Supplementary Data 

# Topoisomerase II minimizes DNA entanglements by proofreading DNA topology after DNA strand passage

## Supplementary Data

files

**Files in this Data Supplement:**

- Supplementary Data - pdf file
