# Supplementary material for: Topoisomerase II minimizes DNA entanglements by proofreading DNA topology after DNA strand passage
Source: Nucleic Acids Res. 2013 Oct 31;42(3):1821–30. doi: 10.1093/nar/gkt1037 (PMC3919613; doi:10.1093/nar/gkt1037)
Supplement: Supplementary Data [file supp_gkt1037_nar-01922-f-2013-File008.pdf]

<u>**SUPPLEMENTARY DATA**</u>

Topoisomerase II minimizes DNA entanglements by proofreading DNA topology after DNA strand passage

Belén Martínez-García, Xavier Fernández, Ofelia Díaz-Ingelmo, Antonio Rodríguez-Campos, Chaysavanh Manichanh and Joaquim Roca*

Instituto de Biología Molecular de Barcelona (IBMB); Consejo Superior de Investigaciones Científicas (CSIC); Barcelona, 08028; Spain.

* To whom correspondence should be addressed.
Tel: +34 934020117; Fax: +34 934034979; Email: joaquim.roca@ibmb.csic.es

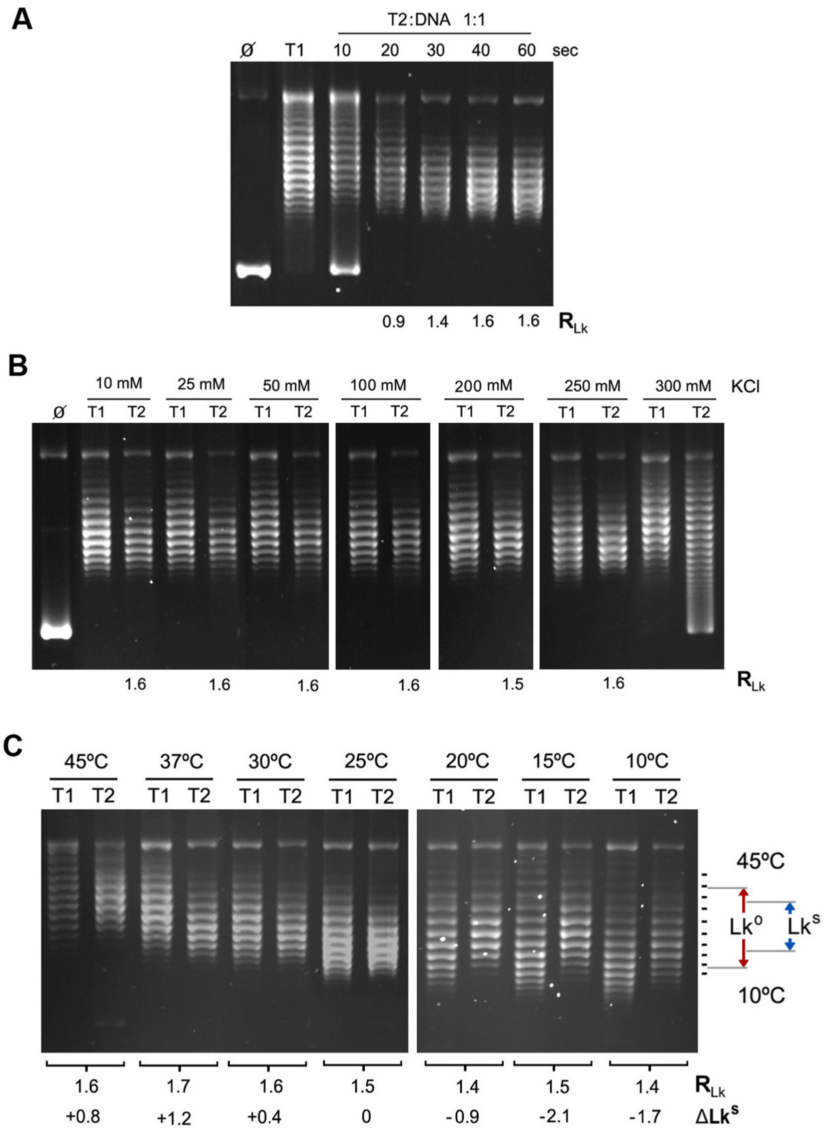

Figure S1. Simplification of equilibrium *Lk* distributions in different reaction conditions. A) Time course. Reactions containing a negatively supercoiled 7.9-kb plasmid (Ø) and T1 or T2 (E:DNA molar ratio 1:1) were done at 37ºC and finished at the indicated times (sec). (B) Effect of salt concentration (KCl) on *Lk* distributions produced by *vaccinia virus* topoisomerase I (T1) and *S. cerevisiae* topoisomerase II (T2). Reactions containing a negatively supercoiled 7.9-kb plasmid (Ø) and T1 or T2 (E:DNA molar ratio 0.5:1) were done at 37ºC during 30 min. R_{Lk} values ($<Lk^2>_{T1}/<Lk^2>_{T2}$) calculated for each salt concentration are indicated. (C) Effect of temperature (ºC) on *Lk* distributions produced by T1 and T2. Reactions containing a negatively supercoiled 7.9-kb plasmid (Ø) and T1 or T2 (E:DNA molar ratio 0.5:1, 150 mM KCl) were done at the indicated temperature during 30 min. R_{Lk} values and ΔLk^S values produced at each temperature are indicated. The range of thermal changes of Lk^0 and Lk^S are compared in the rigth (from 10ºC to 45ºC). General reaction settings, DNA electrophoresis and analysis of *Lk* distributions were conducted as described in the methods.

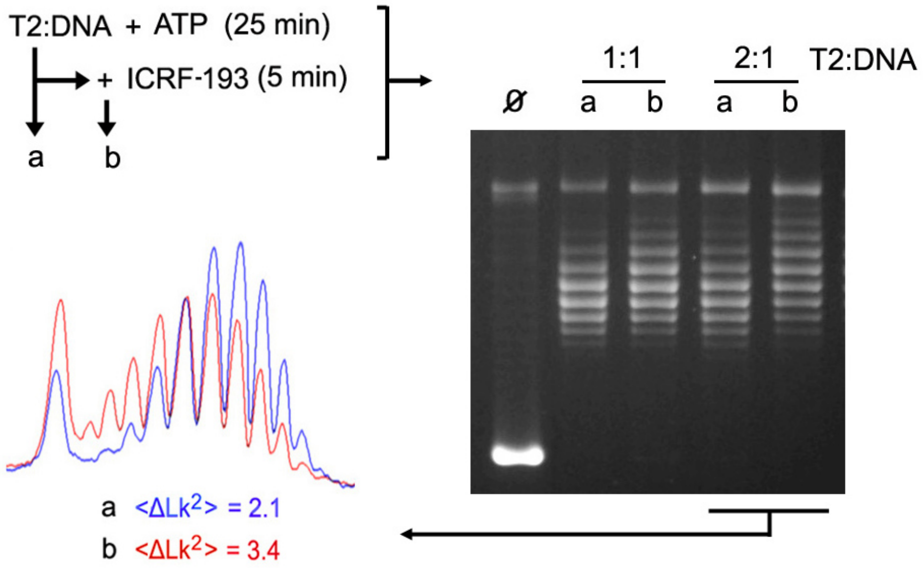

Figure S2. Widening of simplified *Lk* distributions when T-segment backtracking is precluded by locking the N-gate with ICRF-193. A 7.9 kb DNA supercoiled plasmid (Ø) was incubated with T2 (T2:DNA molar ratios 1:1 and 2:1) and ATP (0.1 mM) at 30ºC. After 25 min, one half of each reaction was sampled (lane a) and ICRF-193 (100 µM) was added to the other half for 5 more min (lane b). Gel plots compare the variance of *Lk* distributions ($<\Delta Lk^2>$) produced before and after the addition of ICRF-193 at the T2:DNA molar ratio 2:1. Reaction settings, gel electrophoresis and analyses of *Lk* populations were as described in the methods.

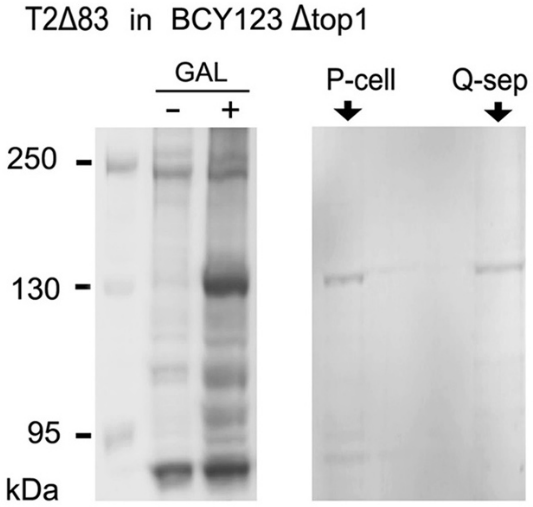

Figure S3. Overproduction of T2Δ83 in yeast cells. Left, *S. cerevisiae* BCY123-*Δtop1* cells were transformed with pGALT2Δ83HMK-His and expression of the T2Δ83 gene construct under the galactose-inducible promoter pGAL1 produced the expected 130 kDa protein. Right, T2Δ83 was purified following the same procedure described for T2. The coomassie stained gel shows pooled fractions containing T2Δ83 after its elution from P-cell and Q-sepharose columns.

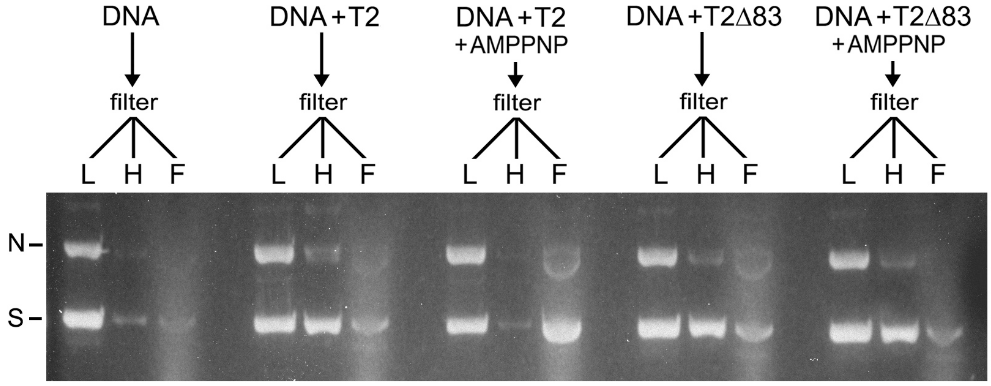

Figure S4. Binding of T2Δ83 to DNA. Samples of a 3 kb DNA plasmid (0.5 pmol) were mixed with no enzyme, with T2 (molar ratio 1:1) or with T2Δ83 (molar ratio 1:1) in a 50 µL volume of buffer L (50 mM Tris–HCl pH 8, 1 mM EDTA, 50 mM KCl, 8 mM $MgCl_2$, 7 mM 2-mercaptoethanol, and 100 µg/ml BSA). Identical mixtures with T2 and T2Δ83 were also prepared and supplemented with AMPPNP (2 mM). Following 30 min incubation at 30°C, each mixture was passed through a GF/C (Whatman) fiberglass filter as described in (29). Successive washes were carried out with buffer L to obtain a low-salt eluate (L), with buffer L plus 1 M NaCl to obtain a high-salt eluate (H), and with 0.5% SDS to obtain a full eluate (F). The agarose gel electrophoresis shown the DNA content found in the eluates. The L eluates reflected free DNA (not bound to topoisomerases), H eluates reflected DNA bound to topoisomerases, F eluates reflected the formation of high salt-resistant topoisomerase:DNA complexes. N, nicked plasmid. S, supercoiled plasmid.
